# Modelling Thermally Induced Non-Equilibrium Gas Flows by Coupling Kinetic and Extended Thermodynamic Methods

**DOI:** 10.3390/e21080816

**Published:** 2019-08-20

**Authors:** Weiqi Yang, Xiao-Jun Gu, David R. Emerson, Yonghao Zhang, Shuo Tang

**Affiliations:** 1 School of Astronautics, Northwestern Polytechnical University, Xi’an 710072, China; 2 Scientific Computing Department, STFC Daresbury Laboratory, Warrington WA4 4AD, UK; 3 James Weir Fluids Laboratory, Department of Mechanical and Aerospace Engineering, University of Strathclyde, Glasgow G1 1XJ, UK

**Keywords:** discrete velocity method, moment method, thermally induced flow, non-equilibrium flow, transition regime

## Abstract

Thermally induced non-equilibrium gas flows have been simulated in the present study by coupling kinetic and extended thermodynamic methods. Three different types of thermally induced gas flows, including temperature-discontinuity- and temperature-gradient-induced flows and radiometric flow, have been explored in the transition regime. The temperature-discontinuity-induced flow case has shown that as the Knudsen number increases, the regularised 26 (R26) moment equation system will gradually loss its accuracy and validation. A coupling macro- and microscopic approach is employed to overcome these problems. The R26 moment equations are used at the macroscopic level for the bulk flow region, while the kinetic equation associated with the discrete velocity method (DVM) is applied to describe the gas close to the wall at the microscopic level, which yields a hybrid DVM/R26 approach. The numerical results have shown that the hybrid DVM/R26 method can be faithfully used for the thermally induced non-equilibrium flows. The proposed scheme not only improves the accuracy of the results in comparison with the R26 equations, but also extends their capability with a wider range of Knudsen numbers. In addition, the hybrid scheme is able to reduce the computational memory and time cost compared to the DVM.

## 1. Introduction

The advent of micro-electro-mechanical systems (MEMS) and the associated fabrication technologies has inspired a renewed impetus in understanding thermally driven flows [1,2,3,4]. Typical examples include thermal transpiration [5,6], radiometric flow [7,8] and Knudsen pumps [5,9]. Since radiometers could also work as Knudsen pumps, transporting gas without any moving parts, they can not only be developed as radiometric actuators for spacecraft attitude control system [10], but also be used for micro-scale gas chromatography and gas separation applications [11,12]. Due to their important applications in industry, many experimental and numerical investigations of thermally induced flow have been carried out [13,14,15].

Numerically, the behaviour of thermally induced non-equilibrium gas flows can be described and modelled from either a microscopic or macroscopic point of view. The Boltzmann equation is the fundamental model for non-equilibrium flows at the microscopic level, which uses a molecular velocity distribution function (VDF) to describe the system state. From a historical perspective, two major categories of approaches have been developed to solve the Boltzmann equation. One is a stochastic approach, such as the direct simulation Monte Carlo (DSMC) method developed by Bird [16], and the other is a deterministic approach. For the latter, one well-known example is the discrete velocity method (DVM) [17], which uses a finite set of discrete velocity points to approximate the continuous molecular velocity space. Due to the multi-dimensional nature of the VDF and the complicated structure of the non-linear collision term, it remains formidable to apply the Boltzmann equation to many practical applications. Hence, extensive work has been devoted to deriving alternative macroscopic modelling strategies for non-equilibrium or rarefied gas flows.

The classical macroscopic equations for gas flows are given by the Navier–Stokes–Fourier (NSF) equations. In association with appropriate velocity-slip and temperature jump wall boundary conditions, they are able to predict certain main features of a flow for simple weakly rarefied problems that are not far away from the equilibrium state, i.e., in the slip regime, but extra care must be taken when thermal effects are present [18]. However, substantial effort is required when the flow departs from the equilibrium state and enters the transition regime. Chapman and Enskog proposed a technique via a formal asymptotic expansion of the molecular VDF in powers of the Knudsen number [19,20]. By truncating the Chapman–Enskog (CE) expansion into different orders, the approach leads to the Euler, NSF, Burnett and super-Burnett equations at the zeroth-, first-, second- and third-order approximation [21], respectively. However, Grad [22] argued that no matter how high the expansion order is, the resulting system will only describe flows that are very close to the continuum solution.

In 1949, Grad [23] developed an alternative approach to derive macroscopic equations via the moment method. In addition to the conservation laws, the governing equations for the stress and heat flux were obtained from the Boltzmann equation. The resulting set of 13 moment equations, closed by expanding the VDF in Hermite polynomials, were denoted as the well-known G13 moment equations [23]. Struchtrup and Torrilhon [24] regularised the G13 equations using a CE-like expansion, and Gu and Emerson [25] and Struchtrup and Torrilhon [26] obtained the wall boundary conditions (WBCs) for the regularised 13 moment equations (R13) based on Maxwell’s kinetic WBC [27]. The R13 equations are able to capture non-equilibrium phenomena at a Knudsen number below 0.25. Furthermore, Gu and Emerson [28] extended the method of moments to derive the regularised 26 (R26) moment equations, which demonstrated their potential as an engineering design tool for non-equilibrium flows in the early transition regime [29,30]. The moment method essentially bridges the gap between conventional hydrodynamic models and kinetic models in the early transition regime, where the NSF and the DVM become either inaccurate or inefficient. In the present study, we couple the R26 moments (at the macroscopic level) with the DVM solver (at the microscopic level) to describe thermally induced non-equilibrium flows.

The remaining part of this paper is organised as follows. We first make an overview of the R26 moment equation system, as described in Section 2. The modelled Boltzmann equation is introduced briefly in Section 3. The hybrid scheme is briefly described in Section 4. Entropy and *H*-theorem will be given in Section 5. Numerical simulations of several types of typical thermally induced non-equilibrium flows (2D thermal cavity flow induced by temperature discontinuity, radiometer flow around a thin plate and cavity flow induced by temperature gradients) are presented and discussed in Section 6 in comparison with the DVM data. A brief summary is finally given in Section 7.

## 2. Extended Thermodynamic Governing Equations

With the traditional thermodynamic variables of velocity, $u_{i},$ temperature, T and density, ρ, the conservation laws for mass, momentum and energy can be expressed as [31]:

(1) $$\left\{ \begin{array}{l} \frac{\partial \rho }{\partial t}+\frac{\partial \rho u_{i}}{\partial x_{i}}=0,\\ \frac{\partial \rho u_{i}}{\partial t}+\frac{\partial \rho u_{i}u_{j}}{\partial x_{j}}+\frac{\partial \sigma_{ij}}{\partial x_{j}}=-\frac{\partial p}{\partial x_{i}},\\ \frac{\partial \rho T}{\partial t}+\frac{\partial \rho u_{i}T}{\partial x_{i}}+\frac{2}{3R}\frac{\partial q_{i}}{\partial x_{i}}=-\frac{2}{3R}\left(p\frac{\partial u_{i}}{\partial x_{i}}+\sigma_{ij}\frac{\partial u_{j}}{\partial x_{i}}\right), \end{array}\right.$$

in which, *t* and $x_{i}=\left(x,y,z\right)$ are the temporal and spatial coordinates, respectively, and any subscript *i*, *j*, *k* represents the usual summation convention. The pressure *p* is related to the temperature *T* by the ideal gas law p=ρRT, where *R* is the gas constant. However, the stress tensor, $\sigma_{ij},$ and the heat flux vector, $q_{i},$ in the set of Equation (1) are unknown. The classic way to close this set of equations is through a CE expansion of the molecular distribution function in terms of Knudsen, Kn, around the Maxwellian [21] to obtain the constitutive relationships for $\sigma_{ij}$ and $q_{i}.$ In the method of moments, Grad [23] derived their governing equations from the Boltzmann equation as:

(2) $$\left\{ \begin{array}{l} \frac{\partial \sigma_{ij}}{\partial t}+\frac{\partial u_{k}\sigma_{ij}}{\partial x_{k}}+\frac{\partial m_{ijk}}{\partial x_{k}}=-A_{\sigma }\frac{p}{\mu }\sigma_{ij}-2p\frac{\partial u_{<i}}{\partial x_{j>}}-\frac{4}{5}\frac{\partial q_{<i}}{\partial x_{j>}}-2\sigma_{k<i}\frac{\partial u_{j>}}{\partial x_{k}},\\ \frac{\partial q_{i}}{\partial t}+\frac{\partial u_{j}q_{i}}{\partial x_{j}}+\frac{1}{2}\frac{\partial R_{ij}}{\partial x_{j}}=-A_{q}\frac{p}{\mu }q_{i}-\frac{5}{2}pR\frac{\partial T}{\partial x_{i}}-\frac{7\sigma_{ik}R}{2}\frac{\partial T}{\partial x_{k}}-RT\frac{\partial \sigma_{ik}}{\partial x_{k}}+\frac{\sigma_{ij}}{\rho }\left(\frac{\partial p}{\partial x_{j}}+\frac{\partial \sigma_{jk}}{\partial x_{k}}\right)\\ \;-\frac{2}{5}\left(\frac{7}{2}q_{k}\frac{\partial u_{i}}{\partial x_{k}}+q_{k}\frac{\partial u_{k}}{\partial x_{i}}+q_{i}\frac{\partial u_{k}}{\partial x_{k}}\right)-\frac{1}{6}\frac{\partial \Delta }{\partial x_{i}}-m_{ijk}\frac{\partial u_{j}}{\partial x_{k}}, \end{array}\right.$$

in which, μ is the dynamic viscosity of the gas. The collision constants, $A_{\sigma }$ and $A_{q},$ are determined by the molecular collision model. The high moments, $m_{ijk},$ $R_{ij}$ and Δ, represent the difference between the true value of the higher moments and their corresponding approximation with the truncated distribution function, $f_{G},$ at the third order in Hermite polynomials. In Grad’s original method [23], such deviations were omitted, so that $m_{ijk}=R_{ij}=\Delta =0$ and the set of Equations (1) and (2) are well known as Grad’s 13 moment equations (G13). Struchtrup and Torrilhon [24] and Struchtrup [31] regularised the G13 moment equations by applying a CE-like expansion and an order-of-magnitude approach and obtained the algebraic constitutive expressions of $m_{ijk},$ $R_{ij}$ and Δ in terms of the derivatives of lower order moments. The regularised G13 moment equations are denoted as the R13 moment equations. Although the R13 moment equations improve the performance of the G13 moment equations significantly, they cannot provide sufficient accurate description of the Knudsen layer [30,32]. To remedy the deficiency of the R13 equations, the governing equations of the high-order moment quantities $m_{ijk},\;R_{ij},\;\Delta$ that can be derived from the Boltzmann equation are employed in the present study. They are [28,29]:

(3) $$\left\{ \begin{array}{l} \frac{\partial m_{ijk}}{\partial t}+\frac{\partial u_{l}m_{ijk}}{\partial x_{l}}+\frac{\partial \phi_{ijkl}}{\partial x_{l}}=-A_{m}\frac{p}{\mu }m_{ijk}-3RT\frac{\partial \sigma_{<ij}}{\partial x_{k>}}-\frac{3}{7}\frac{\partial R_{<ij}}{\partial x_{k>}}+M_{ijk},\\ \frac{\partial R_{ij}}{\partial t}+\frac{\partial u_{k}R_{ij}}{\partial x_{k}}+\frac{\partial \psi_{ijk}}{\partial x_{k}}=-A_{R1}\frac{p}{\mu }R_{ij}-\frac{28}{5}RT\frac{\partial q_{<i}}{\partial x_{j>}}-2RT\frac{\partial m_{ijk}}{\partial x_{k}}-\frac{2}{5}\frac{\partial \Omega_{<i}}{\partial x_{j>}}+{ℜ}_{ij},\\ \frac{\partial \Delta }{\partial t}+\frac{\partial \Delta u_{i}}{\partial x_{i}}+\frac{\partial \Omega_{i}}{\partial x_{i}}=-A_{\Delta 1}\frac{p}{\mu }\Delta -8RT\frac{\partial q_{k}}{\partial x_{k}}+ℵ, \end{array}\right.$$

where the non-linear source terms $M_{ijk},\;{ℜ}_{ij},\;ℵ$ are listed in Appendix A. Similarly, the higher-order moments, $\phi_{ijkl},$ $\psi_{ijk}$ and $\Omega_{i}$ in the set of Equation (3) represent the difference between the true value of the higher moments and their corresponding approximation with the truncated distribution function, $f_{G},$ at the fourth order in Hermite polynomials. A CE-like expansion was employed to obtain the following constitutive relationships [28]:

(4) $$\left\{ \begin{array}{ll} \phi_{ijkl} & =-\frac{4\mu }{A_{\phi 1}\rho }\frac{\partial m_{<ijk}}{\partial x_{l>}}-\frac{4\mu }{A_{\phi 1}p}\left[\frac{3}{7}R_{<ij}\frac{\partial u_{k}}{\partial x_{l>}}+3RT\sigma_{<ij}\frac{\partial u_{k}}{\partial x_{l>}}+m_{<ijk}\frac{\partial RT}{\partial x_{l>}}-\frac{m_{<ijk}}{\rho }\left(\frac{\partial \sigma_{l>m}}{\partial x_{m}}+\frac{\partial p}{\partial x_{l>}}\right)\right]\\ & -\frac{A_{\phi 2}}{A_{\phi 1}}\frac{\sigma_{<ij}\sigma_{kl>}}{\rho },\\ \psi_{ijk} & =-\frac{27\mu }{7A_{\psi 1}\rho }\frac{\partial R_{<ij}}{\partial x_{k>}}-\frac{27\mu }{7A_{\psi 1}\rho }\left(\frac{28}{5}q_{<i}\frac{\partial u_{j}}{\partial x_{k>}}-R_{<ij}\frac{\partial \ln\rho }{\partial x_{k>}}+\frac{R_{<ij}+7RT\sigma_{<ij}}{RT}\frac{\partial RT}{\partial x_{k>}}\right)\\ & -\frac{A_{\psi 2}q_{<i}\sigma_{jk>}+A_{\psi 3}\sigma_{<li}m_{jkl>}}{A_{\psi 1}\rho },\\ \Omega_{i} & =-\frac{7}{3}\frac{\mu }{A_{\Omega 1}\rho }\frac{\partial \Delta }{\partial x_{i}}-\frac{4\mu }{A_{\Omega 1}\rho }\frac{\partial R_{ik}}{\partial x_{k}}+\frac{28\mu }{A_{\Omega 1}p}\left[\frac{2}{3\rho }q_{i}\left(\frac{\partial q_{m}}{\partial x_{m}}+\sigma_{ml}\frac{\partial u_{m}}{\partial x_{l}}\right)-\left(\frac{4}{5}q_{k}\frac{\partial u_{<i}}{\partial x_{k>}}+\sigma_{ik}\frac{\partial RT}{\partial x_{k}}\right)\right]\\ & -\frac{A_{\Omega 2}q_{k}\sigma_{ik}+A_{\Omega 3}m_{ijk}\sigma_{jk}}{A_{\Omega 1}\rho }. \end{array}\right.$$

The closed set of Equations (4) is known as the R26 moment equations.

The values of the collision constants, $A_{\sigma },$ $A_{q},$ $A_{m},$ $A_{R1},$ $A_{R2},$ $A_{\Delta 1},$ $A_{\Delta 2},$ $A_{\phi 1},$ $A_{\phi 2},$ $A_{\psi 1},$ $A_{\psi 2},$ $A_{\psi 3},$ $A_{\Omega 1},$ $A_{\Omega 2}$ and $A_{\Omega 3}$ depend on the molecular collision model adopted and represent the relaxation time-scale for each moment. They are given in Table 1 for the case of the Shakhov model [33] as employed in the present study. The Prandtl number, Pr, is set to be 2/3 for monoatomic gases.

To apply the extended thermodynamic equations to flows in confined geometries, appropriate wall boundary conditions are required to determine a unique solution. Macroscopic wall boundary conditions for confined flows were obtained based on Maxwell’s kinetic wall boundary condition and a fifth-order approximation of the VDF in Hermite polynomials and they are listed in [28]. However, as a truncated VDF is used in the derivation of the set of boundary conditions at a wall where the state of non-equilibrium is strong, the accuracy near the wall is reduced. As a result, it hampers the capability of the moment method. To increase the accuracy of the solution while maintaining a low computational cost, a hybrid algorithm [34], which couples the thermodynamic equations with the kinetic equation in the wall boundary layer, is adopted in the present study.

## 3. Kinetic Equation and the Shakhov Model

From the microscopic view of point, the behaviour of a gas can be described by the kinetic equation:

(5) $$\frac{\partial f}{\partial t}+C_{i}\frac{\partial f}{\partial x_{i}}=-\frac{1}{\tau }\left[f-f^{eq}\right],$$

where $f=f\left(t,x_{i},C_{i}\right)$ is the VDF of gas molecules and $C_{i}$ is the molecular velocity. The mean relaxation time, τ, is evaluated from

(6) $$\tau =\frac{\mu }{p}$$

The Shakhov model [33] is adopted so that the equilibrium VDF, $f^{eq},$ is given by

(7) $$f^{eq}=f^{S}=\frac{\rho }{{\left(2\pi RT\right)}^{3/2}}\exp\left(-\frac{{\left(C_{i}-u_{i}\right)}^{2}}{2RT}\right)\left[1+\left(1-\mathrm{Pr}\right)\frac{\left(C_{i}-u_{i}\right)q_{i}}{5pRT}\left(\frac{{\left(C_{j}-u_{j}\right)}^{2}}{RT}-5\right)\right].$$

Maxwell’s kinetic wall boundary condition [27] is used in association with the kinetic Equation (5). It states that a fraction α of gas molecules undergoes diffusive reflection with a Maxwellian distribution $f_{M}^{w}$ at the temperature of the wall $T^{w}$ while the remaining fraction (1−α) will be reflected specularly. In a frame in which the coordinates are attached to the wall, with $n_{i}$ the normal unit vector of the wall pointing towards the gas such that all molecules with $C_{i}n_{i}<0$ are incident upon the wall and molecules with $C_{i}n_{i}\geq 0$ are emitted by the wall, Maxwell wall boundary condition can be expressed by

(8) $$f^{w}=\{\begin{array}{cc} \alpha f_{M}^{w}+\left(1-\alpha \right)f\left(-C_{i}n_{i}\right), & C_{i}n_{i}\geq 0,\\ f\left(C_{i}n_{i}\right), & C_{i}n_{i}<0, \end{array}$$

with

(9) $$f_{M}^{w}=\frac{\rho^{w}}{{\left(\sqrt{2\pi RT^{w}}\right)}^{3}}exp\left[-\frac{{\left(C_{i}-u_{i}^{w}\right)}^{2}}{2RT^{w}}\right],$$

in which, $u_{i}^{w}$ is the wall velocity and $\rho^{w}$ is the density of the thermalised molecules determined to ensure that no molecules accumulate on the wall.

The modelled Boltzmann equation can be solved alone for the whole flow domain and the solution can be served as the benchmark data. In the hybrid algorithm employed in the present study, the kinetic equation is only solved in the wall boundary layer to provide boundary information for the macroscopic equations solved in the bulk flow domain.

## 4. Hybrid Algorithm of Coupling the Moment Equations and the Kinetic Equation (HYBR)

**Figure 1:**
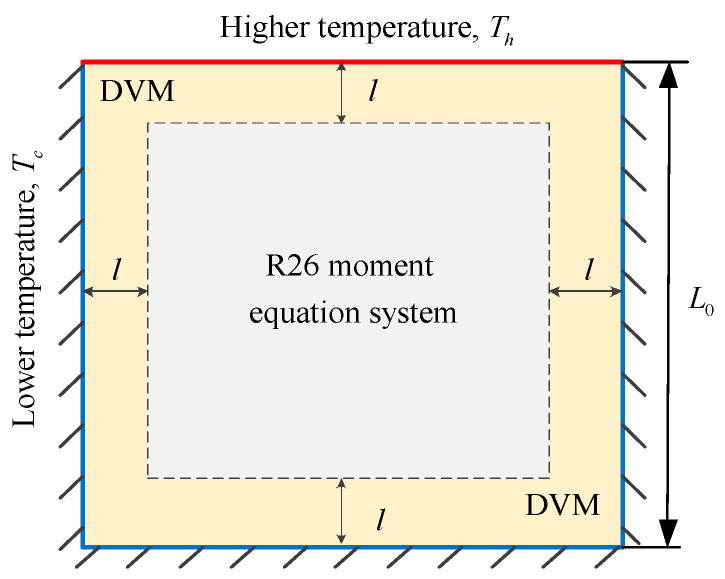
Schematic of the hybrid algorithm for thermally induced cavity flow.

A hybrid algorithm [34] is used to balance the efficiency and the accuracy of a solution, which couples the moment equations and the kinetic equation. Taking a thermally induced cavity flow as an example, we can divide the computational domain into two sub-domains, as shown in Figure 1. In the near-wall subdomain where the non-equilibrium effects are strong, the kinetic equation in association with the Maxwell’s diffusive wall boundary condition is applied, so that accurate description of the flow field can be achieved. The kinetic Equation (5) is solved by the discrete velocity method (DVM) [17,35]. The thickness of the kinetic layer is represented by l. The second-order upwind is used to discretise the spatial derivative in equation. An iteration method is employed as detailed in [35]. As the gas is away from the wall, the R26 moment equations are employed to improve the efficiency. The set of the R26 moment Equations (1)–(3) is solved by a pressure-based numerical algorithm [36] for weakly compressible and low-speed flows. It has been successfully applied in the study of pressure-driven Poiseuille flow [28,37], thermal transpiration flow [5] and gas flows in porous media [38,39].

Once the distribution function, f, is obtained from Equation (5), its moments with respect to the molecular velocity, C, can be determined. For example, the density, ρ, and the momentum, $\rho u_{i}$ can be obtained from

(10) $$\rho =\int fdC\;and\;\rho u_{i}=\int C_{i}fdC.$$

For convenience, the intrinsic or peculiar velocity is introduced as

(11) $$c_{i}=C_{i}-u_{i},$$

so that the moments with respect to $c_{i}$ can be conveniently calculated. A set of N moments are then used to describe the state of the gas through

(12) $$M_{i_{1}i_{2}\cdots i_{N}}=\int c_{i_{1}}c_{i_{2}}\cdots c_{i_{N}}fdC.$$

Any moment can be expressed by its trace and traceless part [31]. For example, the pressure tensor can be separated as follows:

(13) $$p_{ij}=\int c_{i}c_{j}fdC=p\delta_{ij}+p_{\langle ij\rangle }=p\delta_{ij}+\sigma_{ij},$$

where $\delta_{ij}$ is the Kronecker delta, $p=p_{kk}/3$ is the pressure, and $\sigma_{ij}=p_{\langle ij\rangle }$ is the deviatoric stress tensor. The angular brackets are used to denote the traceless part of a symmetric tensor. The temperature, T, is given by thermal energy density as

(14) $$\frac{1}{2}\int c^{2}fdC=\frac{3}{2}\rho RT.$$

The heat flux vector, $q_{i},$ is defined as:

(15) $$q_{i}=\frac{1}{2}\int c^{2}c_{i}fdC.$$

Furthermore, the Grad’s moments, $m_{ijk},$ $R_{ij},$ Δ, $\phi_{ijkl},$ $\psi_{ijk}$ and $\Omega_{i}$ can be evaluated from their definitions by [28].

(16) $$\left\{ \begin{array}{l} m_{ijk}=M_{\langle ijk\rangle }-M_{\langle ijk\rangle }{}_{|f_{G}}=\int c_{<i}c_{j}c_{k>}fdC,\\ R_{ij}=M_{<ij>kk}-M_{<ij>kk|f_{G}}=\int c_{<i}c_{j>}c^{2}fdC-7RT\sigma_{ij},\\ \Delta =M_{rrss}-M_{rrss}{}_{|f_{G}}=\int c^{4}fdC-15pRT,\\ \phi_{ijkl}=M_{\langle ijkl\rangle }-M_{\langle ijkl\rangle }{}_{|f_{G}}=\int c_{\langle i}c_{j}c_{k}c_{l\rangle }fdC,\\ \psi_{ijk}=M_{rr\langle ijk\rangle }-M_{rr\langle ijk\rangle |f_{G}}=\int c_{\langle i}c_{j}c_{k\rangle }c^{2}fdC-9RTm_{ijk},\\ \Omega_{i}=M_{rrssi}-M_{rrssi|f_{G}}=\int c^{4}c_{i}fdC-28RTq. \end{array}\right.$$

These macroscopic quantities not only describe the boundary layer accurately but also provide the boundary information for the R26 moment equations solved away from the wall.

On the other hand, the VDF can be approximated by different order of Hermite polynomials using moments [40] as,

(17) $$f=f_{M}\sum_{n=0}^{\infty }\frac{1}{n!}a_{A}^{\left(n\right)}H_{A}^{\left(n\right)}=f^{eq}\left(a^{\left(0\right)}H^{\left(0\right)}+a_{i}^{\left(1\right)}H_{i}^{\left(1\right)}+\frac{1}{2!}a_{ij}^{\left(2\right)}H_{ij}^{\left(2\right)}+\frac{1}{3!}a_{ijk}^{\left(3\right)}H_{ijk}^{\left(3\right)}+\ldots \ldots \right),$$

where $H_{A}^{\left(n\right)}$ is the Hermite function, and $a_{A}^{\left(n\right)}$ is the corresponding coefficient [40]. The local Maxwellian distribution function is given by

(18) $$f_{M}=\frac{\rho }{{\left(\sqrt{2\pi RT}\right)}^{3}}exp\left[-\frac{c^{2}}{2RT}\right].$$

With the moments available in the R26 moment system, the fifth-order expansion of VDF in Hermite polynomials $f^{\left(5\right)}$ can be expressed by [28]:

(19) $$\begin{array}{ll} f^{\left(5\right)} & =f_{M}[1+\frac{\sigma_{ij}c_{i}c_{j}}{2pRT}+\frac{c_{i}q_{i}}{pRT}\left(\frac{c^{2}}{5RT}-1\right)+\frac{m_{ijk}c_{i}c_{j}c_{k}}{6p{\left(RT\right)}^{2}}+\frac{\phi_{ijkl}c_{i}c_{j}c_{k}c_{l}}{24p{\left(RT\right)}^{3}}+\frac{R_{ij}c_{i}c_{j}}{4p{\left(RT\right)}^{2}}\left(\frac{c^{2}}{7RT}-1\right)\\ & +\frac{\Delta }{8pRT}\left(\frac{c^{4}}{15{\left(RT\right)}^{2}}-\frac{2c^{2}}{3RT}+1\right)+\frac{\psi_{ijk}c_{i}c_{j}c_{k}}{12p{\left(RT\right)}^{3}}\left(\frac{c^{2}}{9RT}-1\right)+\frac{c_{i}\Omega_{i}}{40p{\left(RT\right)}^{2}}\left(\frac{c^{4}}{7{\left(RT\right)}^{2}}-\frac{2c^{2}}{RT}+5\right)]. \end{array}$$

The reconstructed VDF at the coupling interface serves as the boundary condition for the kinetic equation from the bulk flow. In this way, the kinetic equation and the moment equations supply boundary information at the interface for each other and iterate between them until a converged solution is reached.

## 5. Entropy and H-Theorem

Two important features of the solutions to the Boltzmann equation are that the distribution function, f, is non-negative and that the solution must satisfy the H-theorem [41]:

(20) H=−∫flnfdC.

In the method of moment, the VDF is truncated into the fourth- or fifth- order accuracy; hence, it may not satisfy the H-theorem. Struchtrup and Torrilhon have proved that the linearised R13 equations naturally fulfil the H-theorem [26]. In this section, we will explore the validity of the hybrid DVM/R26 method based on its entropy. For equilibrium flow, the value of *H* can be calculated with the equilibrium VDF so that,

(21) $$H^{eq}=-\int f_{M}\ln f_{M}dC=\frac{\rho }{R}\eta +\rho e_{o},$$

where η is the thermodynamic or equilibrium entropy given by [23]

(22) $$\eta =\frac{3}{2}Rln\left(\frac{p}{\rho^{5/3}}\right)$$

and $e_{o}$ is an entropy constant equal to (3/2)(ln2π+1). Therefore, H can be regarded as the entropy for non-equilibrium flows [23,41].

For a homogenous system, the generalised entropy *H* never decreases with time. In the R26 moment equations, with the VDF truncated at the fifth-order accuracy in Hermite polynomials, Gu and Emerson [28] derived an approximated entropy equation for R26 equations where the flow is not far from equilibrium. An alternative way to evaluate the entropy is by directly integrating the reconstructed VDF. In the dimensionless form, $\bar{H},$ it becomes,

(23) $$\bar{H}=-\int {\bar{f}}^{\left(5\right)}\;\ln{\bar{f}}^{\left(5\right)}d\bar{C}.$$

in which, ${\bar{f}}^{\left(5\right)}$ represents the dimensionless form of the reconstructed VDF $f^{\left(5\right)}$ as

(24) $${\bar{f}}^{\left(5\right)}=\frac{{\left(\sqrt{2RT_{ref}}\right)}^{3}}{\rho_{ref}}\;f^{\left(5\right)}\;\mathrm{and}\;\bar{C}=\frac{C}{\sqrt{2RT_{ref}}},$$

where $\rho_{ref}$ and $T_{ref}$ are the reference density and temperature, respectively.

## 6. Numerical Test Cases

In this section, we apply the foregoing hybrid DVM/R26 (HYBR) method to simulate several kinds of thermally induced non-equilibrium flows. In all of the cases, both the DVM and the hybrid DVM/R26 method share the same spatial meshes, and the gas medium is modelled as an argon gas. All of the wall boundaries are treated as diffusive walls. The viscosity is obtained from Sutherland’s law [42]:

(25) $$\mu =\mu_{0}{\left(\frac{T}{T_{0}}\right)}^{1.5}\frac{T_{0}+S}{T+S},$$

where, the Sutherland’s constant *S* for argon is S=144 K and the reference viscosity $\mu_{0}=21.25\times 10^{-6}\mathrm{Pa}\cdot s$ at the temperature $T_{0}=273\;K$.

### 6.1. 2D Thermal Cavity Flow Induced by Temperature Discontinuity

The first case is the flow in a square cavity induced by temperature discontinuities at the cavity boundaries. The geometric configuration is sketched in Figure 1 and the origin of the *x, y* coordinates sits at the centre of the cavity. The characteristic flow length is defined as the side length of the square cavity, which is $L_{0}=10^{-5}\;m$. The temperature on the top wall is maintained at $T_{h}=400\;K$, while on other walls it is maintained at a lower temperature $T_{c}=200\;K$. The reference temperature is set to be $T_{ref}=300\;K$ and the reference pressure $p_{ref}$ is determined by the Knudsen number, Kn, defined by

(26) $$Kn=\frac{\lambda }{L_{0}},$$

in which, the reference mean free path λ can be calculated from the initial reference pressure $p_{ref}$ and viscosity $\mu_{ref}$ at $T_{ref}$ by

(27) $$\lambda =\frac{\mu_{ref}}{p_{ref}}\sqrt{\frac{\pi RT_{ref}}{2}}.$$

**Figure 2:**
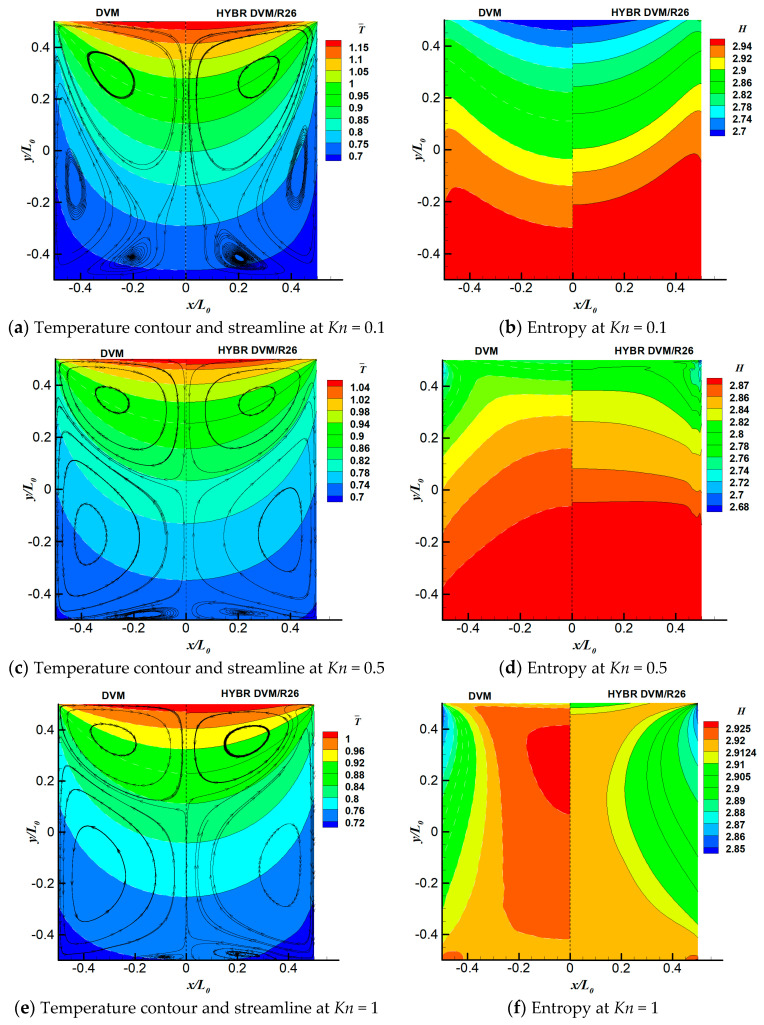
Temperature field, $\bar{T}=T/T_{ref}$ streamlines and system entropy of the temperature-discontinuity-induced flow case at different Knudsen numbers: (**a**,**b**) *Kn* = 0.1, (**c**,**d**) *Kn* = 0.5, (**e**,**f**) *Kn* = 1. **Left panel**: temperature field and streamlines. **Right panel**: system entropy. In each sub-figure, left and right half are results using the DVM and Hybrid DVM/R26 method, respectively.

We consider three Knudsen numbers in this case, i.e., *Kn* = 0.1, 0.5 and 1. For all of the cases, the discrete velocity space is discretised in the range of ${\left[-6\sqrt{2RT_{ref}},6\sqrt{2RT_{ref}}\right]}^{3}$ with 64×64×24 non-uniform points. The physical space is meshed with 101×101 non-uniform points. For the hybrid DVM/R26 method, we apply 5 grid points near the wall boundary where the ratio of the thickness of the kinetic layer *l* to the characteristic length $L_{0}$ is $l/L_{0}=3.64\%$. The temperature contours and the streamlines, as well as the system entropies at different Knudsen numbers are shown in Figure 2. On the left side of each plot are the results from the DVM solution of the kinetic equation. The hybrid DVM/R26 results are presented on the right side of each plot.

As indicated in Figure 2, the overall agreement between the DVM and the hybrid DVM/R26 results, especially in terms of the temperature field, $\bar{T}=T/T_{ref}$, is good. When *Kn* = 0.1, the gas molecules travel from the upper hot wall directly to the bottom wall, and there are two more vortices near the left and right walls. As the Knudsen number increases, two vortices closer to the upper wall shrink. The vortices near the side walls begin to dissolve the vortices on the bottom wall; as a result, the side wall vortices grow larger, and the bottom wall vortices become smaller from *Kn* = 0.5 to 1. Both the DVM and the hybrid DVM/R26 method can capture these vortices accurately. When *Kn* = 1, the hybrid DVM/R26 method slightly overpredicts the size of the vortices near the bottom wall and slightly underpredicts the size of the vortices near the side walls.

Figure 2b shows that the entropy contours calculated from the DVM and hybrid DVM/R26 methods agree well with each other. The entropy at the bottom wall is higher than that in the upper wall. That is because the gas molecules travel away from the upper wall, and they accumulate near the bottom wall region. When *Kn* = 0.5, the hybrid DVM/R26 method slightly overpredicts the entropies near the side wall. As the Knudsen number further increases, i.e., *Kn* = 1, the vortices near the side walls and bottom wall merge together, which drive more gas molecules from the upper and lower walls to the centre of the cavity, causing the entropy there to be larger than that near the bottom wall. Both the DVM and the hybrid DVM/R26 methods can reproduce the entropies accurately.

**Figure 3:**
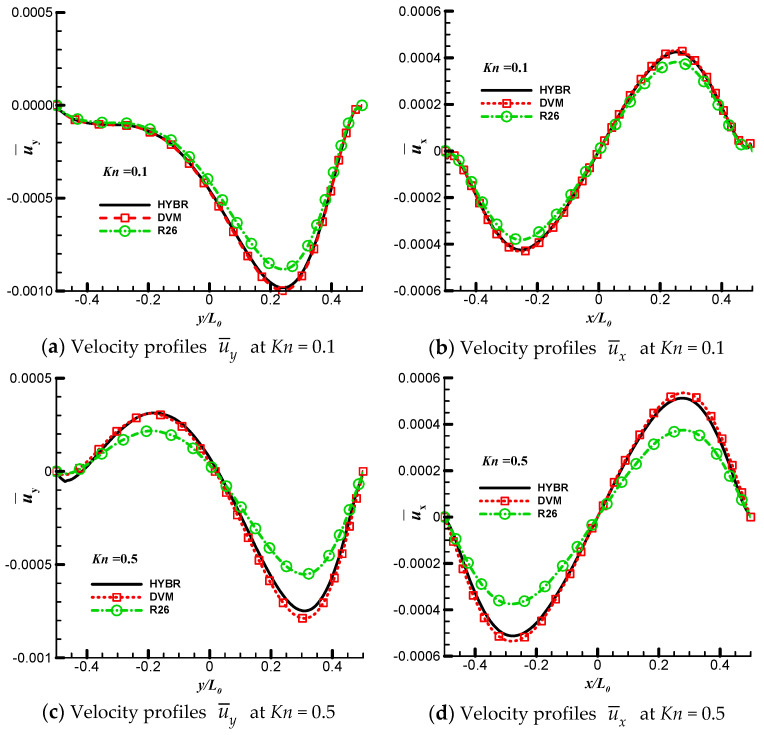

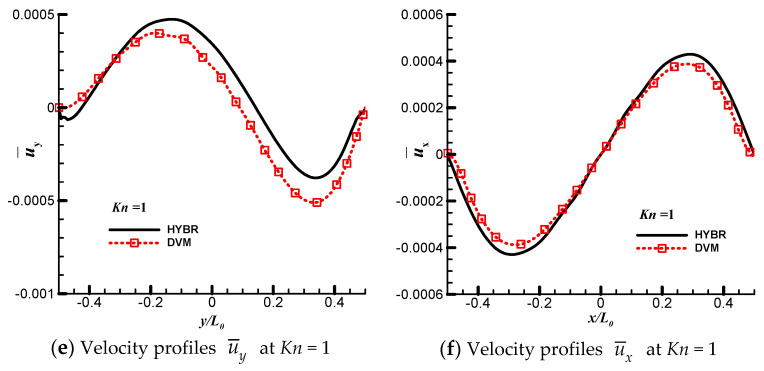
Velocity profiles of the temperature-discontinuity-induced flow case. (**a**,**c**,**e**) Normalised velocity profiles ${\bar{u}}_{y}=u_{y}/\sqrt{2RT_{ref}}$ along the vertical centre line of the cavity at *Kn* = 0.1, 0.5 and 1, respectively. (**b**,**d**,**f**) Velocity profiles ${\bar{u}}_{x}=u_{x}/\sqrt{2RT_{ref}}$ along the horizontal centre lines of the cavity at *Kn* = 0.1, 0.5 and 1, respectively. The black line and ‘HYBR’ are results obtained from the hybrid DVM/R26 method; the red dot and square and ‘DVM’ are results obtained from the DVM.

The normalised velocity profiles along the centre lines of the cavity are shown in Figure 3a–f. From these figures, we can see that the hybrid DVM/R26 results agree well with the DVM results when the Knudsen number is below 0.5. In contrast, the R26 moment equations in association with their wall boundary conditions underpredict the absolute value of maximum velocity by about 9.5% and 28.5% at *Kn* = 0.1 and 0.5, respectively. The hybrid scheme improves the accuracy of the results in comparison with the original R26 moment equations. When *Kn* = 1, it is very difficult for the R26 moment equations to find a converged solution, and the hybrid DVM/R26 method overpredicts the velocity by about 9.5% at the centre of the cavity.

Since we only applied 5 grid points in the computational kinetic layer in the hybrid DVM/R26 method, the computational costs can be significantly reduced. For this case, all tests are done on a single processor. The computational cost, in terms of the computational memory and time cost, has been given in Table 2. The convergence criterion for the steady-state is defined by

(28) $$E\left(n+1\right)=\frac{\sum \left|u^{n+1}-u^{n}\right|}{\sum u^{n+1}}<10^{-6},$$

where *n* and *n* + 1 stand for the *n-*th and (*n* + 1)-th iterations.

It is clear to see that the hybrid DVM/R26 method has the ability to save tremendous memory usage by about 70.1% in comparison with the DVM, and thus save the computational time cost, especially at the low Knudsen numbers. As the Knudsen number increases, the convergence rate of implicit DVM also increases rapidly. Therefore, our newly developed hybrid DVM/R26 method is suitable for Kn≤1, especially when the computational domain is much larger than the near-wall region. When Kn>1, the implicit DVM is fast enough to get the steady-state solutions.

### 6.2. Radiometric Flow

**Figure 4:**
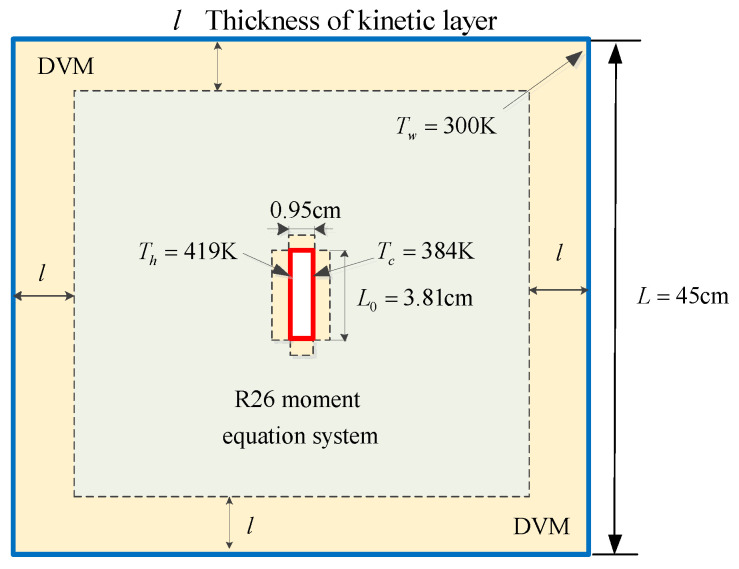
Geometry configuration and the hybrid arrangement of the radiometric flow in a closed chamber.

In this case, we investigate another thermally induced flow, i.e., radiometric flow, which is generated by a small plate with differentially heated sides placed in a chamber. The force acting on the small plate is called radiometric force known to be the driven mechanism of the radiometer [1]. The flow configuration as well as the hybrid arrangement is sketched in Figure 4. The hot small plate with a dimension of $3.81\times 0.95{\;\mathrm{cm}}^{2}$ sits at the geometric centre of the chamber (x=0, y=0). The temperatures of the left and right surface of the plate are kept at $T_{h}=419\;K$ and $T_{c}=384\;K$, respectively. The upper and lower sides of the plate are maintained at 400K. The size of the outer chamber is $45\times 45\;{\mathrm{cm}}^{2}$, and the temperature is kept at $T_{w}=300\;K$. The reference length and the reference temperature are defined as the height of the plate $L_{0}=3.81\;\mathrm{cm}$ and the temperature of the outer chamber $T_{ref}=300\;K$, respectively. A non-uniform mesh with 63,800 cells is used and it is refined near the surface of the plate. The discrete velocity space is discretised in the range of ${\left[-6\sqrt{2RT_{ref}},6\sqrt{2RT_{ref}}\right]}^{3}$ with 64×64×24 non-uniform points. Three Knudsen numbers, i.e., *Kn* = 0.1, 0.5 and 1, are calculated. Five grid points are employed in the kinetic layer in the hybrid DVM/R26 method with $l/L_{0}=0.098$.

**Figure 5:**
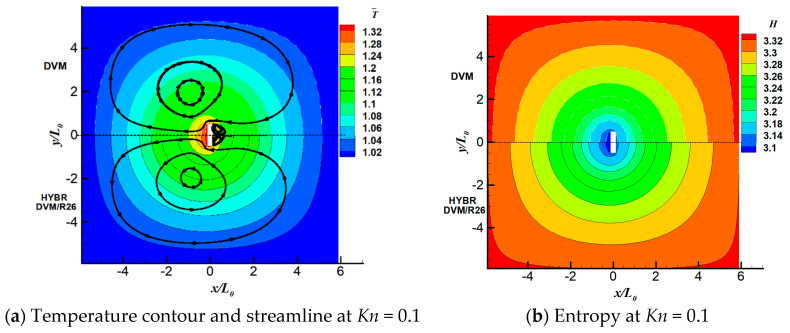

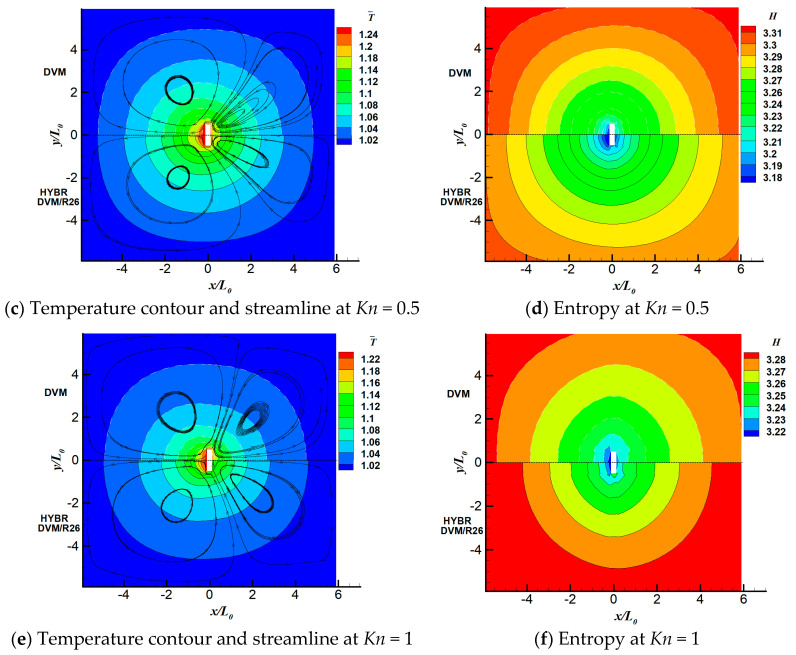
Temperature field, $\bar{T}=T/T_{ref}$, streamlines and entropy field of the radiometric flow case. In each sub-figure, upper and lower half are the results using the DVM and Hybrid DVM/R26 method, respectively.

The temperature fields $\bar{T}=T/T_{ref}$ and streamlines, as well as the entropy fields predicted by both the DVM and hybrid DVM/R26 methods are presented in Figure 5. For the case of *Kn* = 0.1 and 0.5, the overall agreements between the DVM and the hybrid DVM/R26 results are very good in terms of both temperature and velocity fields. The hybrid method slightly underpredicts the size of the vortex near the right side of the hot plate at *Kn* = 0.1. In terms of the system entropy, the hybrid DVM/R26 method is able to reproduce the accurate entropies with the truncated VDF $f^{\left(5\right)}$ at *Kn* = 0.1 and 0.5. It slightly underpredicts the entropy by about 0.3%. When *Kn* = 1, the gas is well into the transition regime and the VDF is far away from the equilibrium, the hybrid scheme slightly overpredicts the entropy by about 0.3%. Shown in Figure 5a,c,e are four vortices generated near four corners of the hot plate. The two vortices near the right side of the plate become larger and the other two vortices become smaller as the Knudsen number increases.

**Figure 6:**
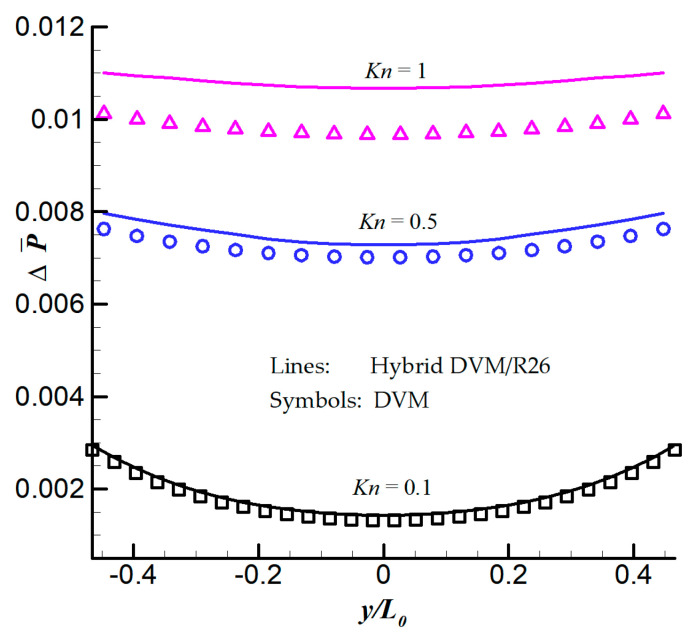
Distribution of normal pressure (stress) difference between the hot and cold sides of the plate along the vertical direction. Lines: results obtained from the hybrid DVM/R26 method. Symbols: results obtained from the DVM.

Figure 6 presents the dimensionless normal pressure (normal stress) difference between the left and right side of the plate along the vertical direction, defined in Equation (29), which is the main contribution to the radiometric force as having been analysed [7],

(29) $$\Delta \bar{P}=\frac{\left[{\left(p+\sigma_{nn}\right)|}_{left}-{\left(p+\sigma_{nn}\right)|}_{right}\right]}{p_{0}},$$

where the subscript *nn* stands for the normal component of the stress tensor relative to the wall.

At the lower Knudsen number, i.e., *Kn* = 0.1, the left/right pressure difference takes larger value near the top and bottom of the plate and lower value near the centre of the plate. A good agreement can be found between the DVM and the hybrid DVM/R26 method at Knudsen number below a value of 0.5. When *Kn* = 1, the distribution of the normal pressure difference is nearly uniform along the plate surface in the vertical direction, and the hybrid DVM/R26 method overpredicts the pressure difference by about 10%. For this case, all tests are done on a single processor, and the computational memory and time cost are listed in Table 3. The convergence criterion for the steady-state is defined by Equation (28). We can see that the hybrid DVM/R26 method has the ability to save the computational memory and time cost. It is because we only use 5 grid points in each kinetic layer in the hybrid DVM/R26 scheme, so that the Boltzmann model equation is solved only in a small region.

### 6.3. 2D Thermal Cavity Flow Induced by the Temperature Gradients

**Figure 7:**
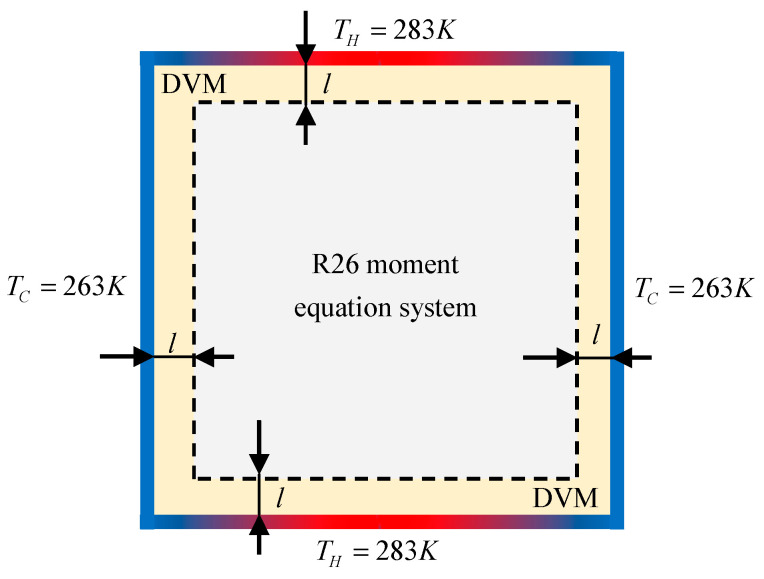
Numerical setup and hybrid arrangement for thermally driven cavity flow.

The last case is the thermal cavity flow induced by temperature gradients at wall, which is also a benchmark case to evaluate the accuracy of the numerical scheme. The computational domain is $10^{-5}\times 10^{-5}m^{2}$ square partitioned by structured rectangular mesh as shown in Figure 7. The left and right walls are maintained at constant temperature $T_{C}=263\;K$. At the top and bottom walls, we introduce a linearly increasing temperature (from $T_{C}=263\;K$ to $T_{H}=283\;K$) in left half of domain, and a linearly decreasing temperature (from $T_{H}$ to $T_{C}$) in the right half. All the walls are treated as diffusive boundaries. The reference mean free path λ is calculated from the initial uniform density. The reference temperature $T_{ref}$ and the characteristic length $L_{0}$ are set to be 273K and $10^{-5}$ m, respectively.

**Figure 8:**
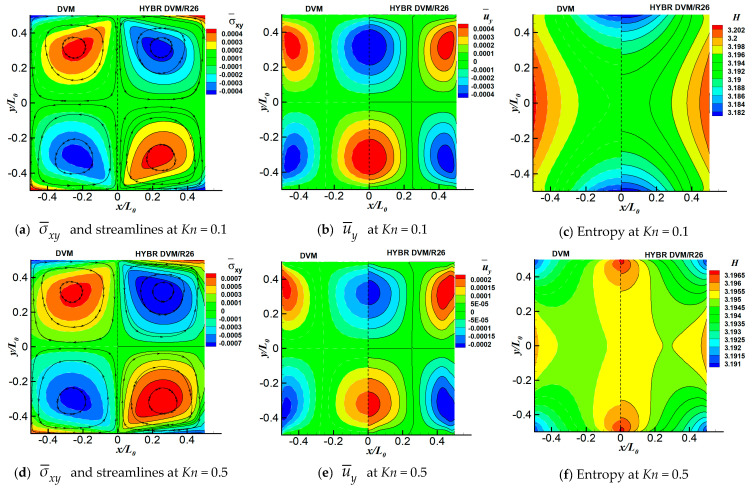

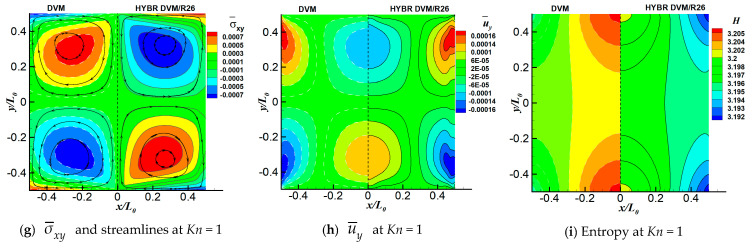
Comparison of ${\bar{\sigma }}_{xy}=\sigma_{xy}/\left(2\rho_{ref}RT_{ref}\right)$ and ${\bar{u}}_{y}=u_{y}/\sqrt{2RT_{ref}}$ contours, streamlines and system entropy *H* of the temperature-gradient-induced flow case at different Knudsen numbers: (**a**–**c**) *Kn* = 0.1, (**d**–**f**) *Kn* = 0.5, and (**g**–**i**) *Kn* = 1. In each sub-figure, left and right half are results using the DVM and Hybrid DVM/R26 method, respectively.

**Figure 9:**
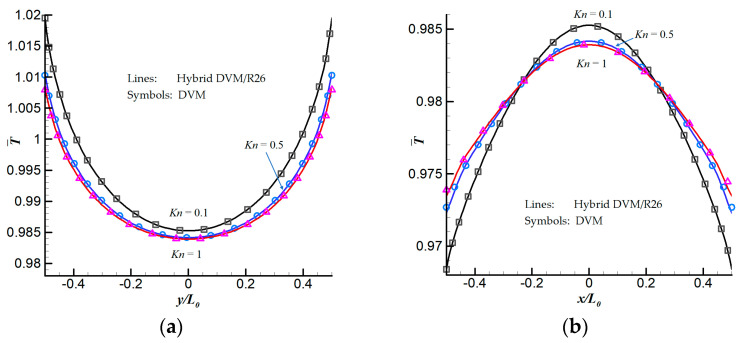
Temperature profiles $\bar{T}=T/T_{ref}$ along the vertical centre (**a**) and the horizontal centre lines (**b**) with different Knudsen numbers. Lines: results obtained from the hybrid DVM/R26 method. Symbols: results obtained from the DVM.

Three cases corresponding to *Kn* = 0.1, *Kn* = 0.5 and *Kn* = 1.0 are computed. For all of these cases, the spatial space is discretised with 101×101 uniform points, and the discrete velocity space is discretised in the range of ${\left[-6\sqrt{2RT_{ref}},\;6\sqrt{2RT_{ref}}\right]}^{3}$ with 64×64×24 non-uniform points. For the hybrid DVM/R26 method, 5 grid points are used in each kinetic layer near the wall boundary with $l/L_{0}=3.64\%$. The entropy in the hybrid DVM/R26 method is calculated from Equation (23). The comparison of the DVM and the hybrid DVM/R26 method on temperature-gradient-induced thermal cavity flow are shown in Figure 8. In addition, the non-dimensional temperature profiles $\bar{T}=T/T_{ref}$ along the vertical and horizontal centre lines are presented in Figure 9.

For the cases of *Kn* = 0.1, 0.5 and 1.0, the hybrid DVM/R26 results are compared with DVM solutions. The overall agreement between the two approaches, especially in terms of the temperature field, is very good. As indicated in Figure 8a,d,g, four vortices are generated with two of them rotating counter-clockwise at the lower left and upper right of the cavity, and another two vortices rotating clockwise at the upper left and lower right of the cavity. As a consequence, the maximum and minimum stresses appear at the centre of clockwise and counter-clockwise vortices, respectively. The absolute values of velocities near the edge of the vortices are higher than that in the centre of the vortices. Both the DVM and the hybrid DVM/R26 methods have the ability to capture these four vortices and flow parameters accurately. It is found in Figure 9 that from the regions near solid walls to the cavity centre, the gas temperature increases along horizontal lines, while it decreases along vertical lines. The maximum temperature value decreases as the degree of rarefaction increases. It is because both the collisions among gas molecules and the interactions between hot wall and gas molecules become weak when the gas is far away from the equilibrium state.

**Figure 10:**
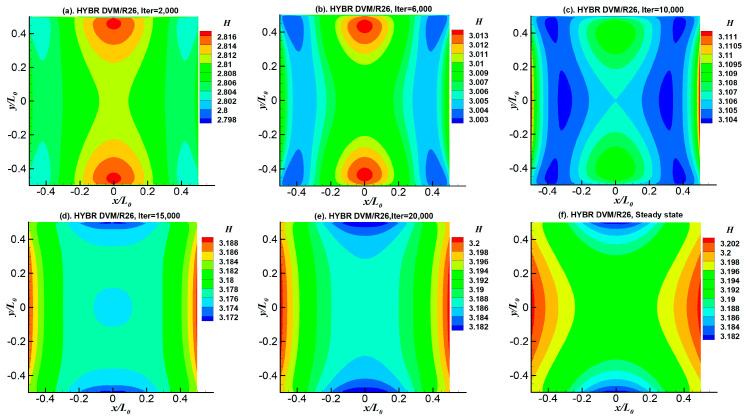
Iteration history of entropy of the temperature-gradient-induced flow case at *Kn* = 0.1.

In terms of the system entropy, it is very interesting to note that, unlike the other flow properties, the distributions of entropies are totally different between the lower and higher Knudsen numbers. We have tracked the iteration history of the system entropy to investigate its fundamental characteristics in detail, as shown in Figure 10. As indicated in Figure 8c,f,i and Figure 10, for all of the cases, the maximum value of entropy first appears near the centre of the upper and lower walls. Meanwhile, the minimum value of entropy first occurs at the four corners. When the Knudsen number is above 0.5, the variations of the overall entropy distribution contours with respect to the iterations are small. Therefore, the basic shapes of the steady-state entropy contours are similar to those of their initial contours. However, when *Kn* = 0.1, the maximum values of entropies first appear near the centre of upper and lower walls, and then they move to the centre of the cavity. In this period, the energy transfers from the hot upper and lower walls to the centre, and produces the system entropy, as indicated in Figure 10b. After the left and right cold walls absorb the energy, the values of entropy near the side walls increase subsequently. With strong gas–wall interactions and intensive molecular collisions, more energy can be transferred directly from the centre of the upper and lower hot walls to the side walls, which lead to higher values of entropies near the side wall.

For this case, both the DVM and the hybrid DVM/R26 method are simulated on a single processor. The computational costs for this case are listed in Table 4. The convergence criterion for the steady-state is defined by Equation (28). As expected, the hybrid DVM/R26 method is able to save the memory cost by about 68.5%, and thus reduce the computational time subsequently.

## 7. Conclusions

In the present study, the application of coupling macro- and microscopic approaches for simulating thermally induced non-equilibrium flows has been explored. The R26 moment equation system is employed at the macroscopic level, meanwhile the Boltzmann model equation associated with the DVM are used to describe the gas dynamics at the microscopic level. Three types of thermally induced flows have been investigated with different Knudsen numbers and the results have been validated using DVM results. The simulation results show that the hybrid DVM/R26 approach can be faithfully used for thermally induced non-equilibrium low-speed flows. Since we only solve the Boltzmann model equation in the near-wall regions, tremendous computational memory and time can be saved in comparison with the DVM. The entropy fields also show that the reconstructed VDF $f^{\left(5\right)}$ is able to yield accurate results when the Knudsen number is less than unity. It is also interesting to find that, unlike the other flow parameters, the distributions of system entropy present totally different characteristics between the lower and higher Knudsen numbers in the temperature gradient cases.

## Figures and Tables

**Table 1:** Collision constants in the moment equations for Shakhov model.

$A_{\sigma }$	$A_{q}$	$A_{m}$	$A_{R1},$ $A_{R2}$	$A_{\Delta 1},A_{\Delta 2}$	$A_{\phi 1},A_{\phi 2}$	$A_{\psi 1},A_{\psi 2},A_{\psi 3}$	$A_{\Omega 1},A_{\Omega 2},A_{\Omega 3}$
1	Pr	1	1, 0	1, 0	1, 0	1, 0, 0	1, 0, 0

**Table 2:** Comparison of computational cost of the temperature-discontinuity-induced cavity case.

	Computational Memory (GB)	Computational Time (Minutes)		
		*Kn* = 0.1	*Kn* = 0.5	*Kn* = 1.0
DVM	11.80	452	204	128
Hybrid DVM/R26	3.53	55	78	96

**Table 3:** Comparison of computational cost of the radiometric flow case.

	Computational Memory (GB)	Computational Time (Minutes)		
		*Kn* = 0.1	*Kn* = 0.5	*Kn* = 1.0
DVM	42.50	983	503	324
Hybrid DVM/R26	10.01	168	243	289

**Table 4:** Comparison of computational cost of the temperature-discontinuity-induced cavity case.

	Computational Memory (GB)	Computational Time (Minutes)		
		*Kn* = 0.1	*Kn* = 0.5	*Kn* = 1.0
DVM	11.22	512	237	156
Hybrid DVM/R26	3.53	68	93	126

## Appendix A. Source Terms in the Moment Equation (3)

(A1) $$\left\{ \begin{array}{l} M_{ijk}=-3\sigma_{<ij}\frac{\partial RT}{\partial x_{k>}}+\frac{3\sigma_{<ij}}{\rho }\left(\frac{\partial p}{\partial x_{k>}}+\frac{\partial \sigma_{k>m}}{\partial x_{m}}\right)-\frac{12}{5}q_{<i}\frac{\partial u_{j}}{\partial x_{k>}}-3m_{m<ij}\frac{\partial u_{k>}}{\partial x_{m}},\\ {ℜ}_{ij}=-A_{R2}\frac{p}{\mu }\frac{\sigma_{k<i}\sigma_{j>k}}{\rho }+\left(\frac{8}{3}RT\sigma_{ij}-\frac{2}{7}R_{ij}\right)\frac{\partial u_{k}}{\partial x_{k}}-\frac{4}{7}\left(7RT\sigma_{k<i}+R_{k<i}\right)\left(\frac{\partial u_{j>}}{\partial x_{k}}+\frac{\partial u_{k}}{\partial x_{j>}}\right)\\ \;-2R_{k<i}\frac{\partial u_{j>}}{\partial x_{k}}+\frac{28}{5}\frac{q_{<i}}{\rho }\frac{\partial \sigma_{j>k}}{\partial x_{k}}+\frac{28}{5}RTq_{<i}\left(\frac{\partial p}{p\partial x_{j>}}-2\frac{\partial T}{T\partial x_{j>}}\right)+2\frac{m_{ijk}}{\rho }\frac{\partial \sigma_{kl}}{\partial x_{l}}\\ \;+m_{ijk}\left(\frac{2}{\rho }\frac{\partial p}{\partial x_{k}}-9\frac{\partial RT}{\partial x_{k}}\right)+\frac{14}{3}\frac{\sigma_{ij}}{\rho }\left(\frac{\partial q_{m}}{\partial x_{m}}+\sigma_{ml}\frac{\partial u_{m}}{\partial x_{l}}\right)-\frac{14}{15}\Delta \frac{\partial u_{<i}}{\partial x_{j>}}-2\phi_{ijkl}\frac{\partial u_{k}}{\partial x_{l}},\\ \;ℵ=-A_{\Delta 2}\frac{p}{\mu }\frac{\sigma_{kj}\sigma_{jk}}{\rho }-\frac{4}{3}\Delta \frac{\partial u_{k}}{\partial x_{k}}-4\left(2RT\sigma_{kl}+R_{kl}\right)\frac{\partial u_{k}}{\partial x_{l}}+8\frac{q_{k}}{\rho }\left(\frac{\partial \sigma_{kl}}{\partial x_{l}}\right)+RTq_{k}\left(8\frac{\partial p}{p\partial x_{k}}-28\frac{\partial T}{T\partial x_{k}}\right). \end{array}\right.$$
